# Consensus Statement for Protocols of Factorial Randomized Trials: Extension of the SPIRIT 2013 Statement

**DOI:** 10.1001/jamanetworkopen.2023.46121

**Published:** 2023-12-01

**Authors:** Brennan C. Kahan, Sophie S. Hall, Elaine M. Beller, Megan Birchenall, Diana Elbourne, Edmund Juszczak, Paul Little, John Fletcher, Robert M. Golub, Beatriz Goulao, Sally Hopewell, Nazrul Islam, Merrick Zwarenstein, An-Wen Chan, Alan A. Montgomery

**Affiliations:** MRC Clinical Trials Unit at UCL, London, United Kingdom; Nottingham Clinical Trials Unit, School of Medicine, University of Nottingham, Nottingham, United Kingdom; Institute for Evidence-Based Healthcare, Bond University, Robina, Australia; Nottingham Clinical Trials Unit, School of Medicine, University of Nottingham, Nottingham, United Kingdom; London School of Hygiene and Tropical Medicine, London, United Kingdom; Nottingham Clinical Trials Unit, School of Medicine, University of Nottingham, Nottingham, United Kingdom; Primary Care Research Centre, School of Primary Care, Population Sciences and Medical Education, Faculty of Medicine, University of Southampton, Southampton, United Kingdom; The BMJ, London, United Kingdom; Department of Medicine, Northwestern University Feinberg School of Medicine, Chicago, Illinois; Health Services Research Unit, University of Aberdeen, Aberdeen, Scotland; Oxford Clinical Trials Research Unit, University of Oxford, Oxford, United Kingdom; Primary Care Research Centre, School of Primary Care, Population Sciences and Medical Education, Faculty of Medicine, University of Southampton, Southampton, United Kingdom; The BMJ, London, United Kingdom; Centre For Studies in Family Medicine, Schulich School of Medicine and Dentistry, Western University, London, Canada; Women’s College Research Institute, University of Toronto, Toronto, Canada; Nottingham Clinical Trials Unit, School of Medicine, University of Nottingham, Nottingham, United Kingdom

## Abstract

**Importance:**

Trial protocols outline a trial’s objectives as well as the methods (design, conduct, and analysis) that will be used to meet those objectives, and transparent reporting of trial protocols ensures objectives are clear and facilitates appraisal regarding the suitability of study methods. Factorial trials, in which 2 or more interventions are assessed in the same set of participants, have unique methodological considerations. However, no extension of the Standard Protocol Items: Recommendations for Interventional Trials (SPIRIT) 2013 Statement, which provides guidance on reporting of trial protocols, for factorial trials is available.

**Objective:**

To develop a consensus-based extension to the SPIRIT 2013 Statement for factorial trials.

**Evidence Review:**

The SPIRIT extension for factorial trials was developed using the Enhancing the Quality and Transparency of Health Research (EQUATOR) methodological framework. First, a list of reporting recommendations was generated using a scoping review of methodological articles identified using a MEDLINE search (inception to May 2019), which was supplemented with relevant articles from the personal collections of the authors. Second, a 3-round Delphi survey (January to June 2022, completed by 104 panelists from 14 countries) was conducted to assess the importance of items and identify additional recommendations. Third, a hybrid consensus meeting was held, attended by 15 panelists to finalize selection and wording of the checklist.

**Findings:**

This SPIRIT extension for factorial trials modified 9 of the 33 items in the SPIRIT 2013 checklist. Key reporting recommendations were that the rationale for using a factorial design should be provided, including whether an interaction is hypothesized; the treatment groups that will form the main comparisons should be identified; and statistical methods for each main comparison should be provided, including how interactions will be assessed.

**Conclusions and Relevance:**

In this consensus statement, 9 factorial-specific items were provided that should be addressed in all protocols of factorial trials to increase the trial’s utility and transparency.

## Introduction

Trial protocols describe the study rationale, objectives, and proposed methods, including the statistical analysis.^1,2^ Trial protocols are used by study investigators and staff as a guide to trial implementation, research ethics committees to try to ensure the study is ethical, and journals, regulatory agencies, and reviewers to evaluate the conduct and reporting of trials.^1,2^ To help ensure trial protocols were fit to meet these objectives, the Standard Protocol Items: Recommendations for Interventional Trials (SPIRIT) 2013 Statement was developed.^1,2^ The SPIRIT statement provides a checklist of 33 items to report. SPIRIT focuses mainly on 2-group parallel designs, and although most items will be applicable to more complicated designs, adaptation or additional items may be required.

Factorial trials are trials in which 2 or more interventions are assessed in the same participants within a single study.^3–16^ An example of a 2 × 2 factorial trial with factors A and B is shown in Table 1. Here, participants are allocated to intervention A or its comparator, and also to intervention B or its comparator, meaning participants are assigned to 1 of 4 treatment groups: A alone, B alone, A and B, or neither A nor B (double control). Factorial trials have additional methodological complexities compared with parallel-group designs. They can be used to address different research questions (ie, estimands) (Box) that require different methods. For instance, factorial trials can be used to evaluate multiple interventions in a single trial, or to evaluate whether treatments interact, ie, whether the effect of one treatment depends on whether participants receive the other treatment or not.^10,15,17,18^ Additional complexities include which treatment groups should be included in main comparisons, how potential interactions are to be handled during analysis, and nonconcurrent enrollment of participants.^3,4,6,8,12–15,19^

In this consensus statement, an extension of the SPIRIT 2013 checklist for the reporting of factorial trial protocols is presented.^1,2^ The term *factor* is used to describe each overall intervention and its comparator (eg, factor A comprises A and not A), and *treatment group* is used to describe the unique combinations of factors and levels (eg, A alone, B alone, A and B, and neither A nor B are the 4 treatment groups in a 2 × 2 design). A glossary of key terms is provided in Table 2. This statement focuses on 2 × 2 factorial trials, although reporting recommendations will apply to more complex factorial designs, such as those with more than 2 factors or more than 2 levels per factor.

## Methods

The development of this SPIRIT extension occurred in parallel with the Consolidated Standards of Reporting Trials (CONSORT) reporting guideline extension for factorial trials.^20^ This extension was developed using the Enhancing the Quality and Transparency of Health Research (EQUATOR) methodological framework, and this report follows the Standards for Quality Improvement Reporting Excellence (SQUIRE) reporting guideline.^21^ Full methods are available elsewhere.^22^ We began with a scoping review to create an initial list of reporting recommendations for factorial trial protocols, which included methodological articles published up to May 2019, as well as those from the personal collections of the authors. After compiling a list of recommendations and obtaining funding, we performed a 3-round Delphi survey (January to June 2022) to rate the importance of each item and to receive suggestions for additional items. We then held a hybrid consensus meeting (September 6-7, 2022, attended by 15 panelists) followed by email discussion to reach agreement on the content and wording of the final checklist.

## Results

Table 3 shows the modified checklist for the reporting of factorial trial protocols. It includes 9 items that have been modified from the SPIRIT 2013 Statement.

The scoping review identified 19 recommendations pertinent to factorial trial protocols, which were evaluated in the Delphi survey. Each recommendation was evaluated separately, even if multiple recommendations were relevant to the same SPIRIT item. There were 104 Delphi participants: 60 were statisticians, 25 were clinical trialists, 7 were trial managers, 19 had experience as a chief investigator, 17 had experience as a journal editor, and 2 were patient and public involvement members (participants could select ≥1 role).^22^ Twenty recommendations met the criteria to be evaluated at the consensus meeting (1 recommendation was added in round 2 of the Delphi survey). After the consensus meeting, with further discussions by teleconference and email, the extension checklist was finalized.

Given the variation in terms used to describe factorial trials, the items in this statement have been written to replace the original SPIRIT items. When using the updated checklist, users are advised to refer to definitions of key terms in Table 2.

This report contains brief explanations of the modified items in the SPIRIT factorial extension. Details for interpretation of each item and examples of good reporting will be presented in a separate explanation and elaboration article.

### SPIRIT Checklist Extension for Factorial Trial Protocols

**Item 1. SPIRIT 2013: Descriptive title identifying the study design, population, interventions, and, if applicable, trial acronym**

**Extension for factorial trials: Identification as a factorial randomized trial in the title** | Factorial designs have unique methodological features, so by alerting readers to the design, they may consider implications and potential limitations.^4,6,7,10,23,24^

**Item 6a. SPIRIT 2013: Description of research question and justification for undertaking the trial**

**Extension for factorial trials: Rationale for using a factorial design, including whether an interaction is hypothesized** | Factorial trials can be used to address different research hypotheses (ie, estimands) (Box). For example, they can evaluate more than 1 intervention in a single trial without the need to increase the sample size (often described as *2-in-1 trials*) to evaluate whether interventions interact (ie, whether the effect of treatment A depends on whether patients receive the other factor or not), or to identify the best combination of interventions. Clarifying the reason for using the factorial design, as well as whether an interaction is hypothesized, enables readers to understand the key objectives and as well as the assumptions underpinning the use of the factorial design.^3,6–8,24^

**Item 7. SPIRIT 2013: Specific objectives or hypotheses**

**Extension for factorial trials: A statement of which treatment groups will form the main comparisons** | Factorial trials allow investigators to compare interventions in different ways. For example, in a 2 × 2 factorial trial with factors A and B, the treatment effect for intervention A vs its comparator can be estimated by comparing (1) participants allocated to A vs not A; (2) those allocated to A alone vs neither A nor B; or (3) those allocated to A and B vs B alone. These different comparisons may target different estimands and require different assumptions.^6,8,13^ An estimand describes the treatment effect investigators intend to estimate from the trial.^13,25,26^

**Item 8. SPIRIT 2013: Description of trial design, including type of trial, allocation ratio, and framework**

**Extension for factorial trials: Description of the type of factorial trial (such as a full or partial, number of factors, and levels within each factor)** | Various types of factorial designs can be used. The simplest design is a full factorial design, in which all participants are eligible to be allocated to all combination of factors and factor-levels.^11,27,28^ The fractional factorial designs (in which some combinations of factors are omitted) and partial factorial designs (in which some participants are only eligible to be randomized to certain factors) require different methods.^3,29^

**Item 10. SPIRIT 2013: Inclusion and exclusion criteria for participants**

**Extension for factorial trials: Eligibility criteria for each factor, noting any differences, if applicable** | Differences in eligibility criteria among factors can require modifications to the sample size and analysis and can lead to bias if not handled properly during analysis. Participants who are not eligible for randomization to a specific factor should be omitted from the comparison for that factor (and any assessment of interaction), as their inclusion means the analysis is no longer based on a randomized comparison, which can lead to confounding bias.^3,29^

**Item 14. SPIRIT 2013: Estimated number of participants needed to achieve study objectives and how it was determined**

**Extension for factorial trials: How sample size was determined for each main comparison, including whether an interaction was assumed in the calculation** | The appropriate sample size calculation depends both on the specific rationale for using the factorial design as well as the methodology used to undertake the trial. For instance, trials designed to assess whether interventions interact typically require larger sample sizes than those aiming to assess the effect of each intervention; for 2-in-1 trials, the planned method of analysis (factorial vs multiarm) will affect the required sample size. Furthermore, for some factorial trials, the planned main comparisons may require different sample sizes; this can occur if they are expected to produce different effect sizes, or if the choice of primary outcome varies for each factor.^8,30^

**Item 16a. SPIRIT 2013: Method of generating the allocation and list of any factors for stratification**

**Extension for factorial trials: If applicable, whether participants will be allocated to factors at different time points** | In some factorial trials, participants may be randomized to factors at different time points. For example, they may be randomized for factor A at diagnosis, then for factor B once treatment A is complete. The time point of randomization for each factor informs key design features, such as the baseline period, duration of follow-up, and likelihood of treatments interacting.^4^

**Item 20a. SPIRIT 2013: Statistical methods for analyzing primary and secondary outcomes; reference to where other details of the statistical analysis plan can be found, if not in the protocol**

**Extension for factorial trials: Statistical methods used for each main comparison for primary and secondary outcomes**

- **Whether the target treatment effect for each main comparison pertains to the effect in the presence or absence of other factors**

Understanding the exact treatment effect being estimated is essential to proper interpretation of study results. However, this is not always clear from the study methods alone.^31–33^ A particular issue for factorial trials is that the treatment groups used for comparison are not always the same as those in which there is interest in estimating the treatment effect.^13,34^ For instance, many factorial trials use a factorial analysis to compare groups all A vs all not A for reasons of efficiency, although interest really lies in the effect of A alone vs control (the effect of A in the absence of B), or, alternatively, the effect of A and B vs B alone (the effect of A in the presence of B) if treatment B has been demonstrated to be effective.^13^ A clear description of the target treatment effect, including whether it pertains to the effect in the presence or absence of other factors, allows readers to understand the exact question being addressed.^13,25,31,32^ The target treatment effect is called the *estimand* and should be specified for each comparison.^13,25^

- **Approach to analysis**

Depending on the estimand of interest, different statistical methods can be used to analyze factorial trials. The 2 most common methods of evaluating interventions are factorial (or at-the-margins) analysis^4,6,8,13,35,36^ and multiarm (or inside-the-table) analysis.^4,6–8,12–14,19,23,35,36^ Using Table 1 as an example, in the factorial analysis, all participants allocated to factor A (active A + active B and active A + control B) are compared with all those not allocated to A (control A + active B and control A + control B). In a multiarm analysis, each individual treatment group is compared against a reference (eg, active A + control B, control A + active-B, and active A + active B vs control A + control B). The 2 approaches offer different advantages and require different assumptions (Box).

- **How the approach will be chosen**

Investigators sometimes use an initial test of interaction to decide whether to use a factorial or multiarm analysis. This approach can introduce bias.^19^ As such, it is generally not recommended; however, if this approach is being used, it is important to report this so that readers can understand the statistical implications of the analysis approach.

- **Method(s) used to evaluate statistical interaction(s)**

Evaluating whether treatments interact is typically required in factorial trials, either because analyses rely on the assumption that treatments do not interact, or because the interaction is itself of direct interest.^4,6–8,12,13,24^ Reporting details of how interactions will be evaluated enables readers to understand the appropriateness of methods.

- **Whether factors will be adjusted for each other**

Factorial analyses can be adjusted for whether participants were allocated to the other factors by including a term for this in the statistical model.^4,8,13,30^ This can increase statistical power, and in some cases, failure to adjust for the other factors can introduce bias for some estimands.^13^

- **How nonconcurrent recruitment to factors will be handled**

Nonconcurrent recruitment, in which certain participants are not randomized for some factors (eg, if recruitment to 1 of the factors is paused or terminated), can induce bias if not handled correctly during analysis.^3,29^ Therefore, understanding whether participants not randomized for a factor were excluded from the analysis for that factor is necessary to understand the risk of bias.

**Item 21b. SPIRIT 2013: Description of any interim analyses and stopping guidelines, including who will have access to these interim results and make the final decision to terminate the trial**

**Extension for factorial trials: When applicable, explanation of any interim analyses and stopping guidelines, noting any differences across main comparisons and reasons for differences** | Interim analyses are often used for reasons of safety, efficacy, or futility. Stopping guidelines may be different for each factor.^29^ If 1 factor is stopped before the other, there may be implications for randomization, choice of comparator, or the analysis population.^3,29,37^

## Discussion

The SPIRIT 2013 Statement provides a comprehensive checklist for the reporting of clinical trial protocols, with the aims of facilitating good trial conduct and appraisal by ensuring clarity around the trial’s design, conduct, and analyses.^1,2^ This extension to the SPIRIT 2013 Statement provides guidance on reporting of factorial trial protocols. Clear reporting of factorial trial protocols can help investigators ensure planned trial procedures are clear and comprehensive and facilitate appraisal by readers of the protocols, such as research ethics committees and reviewers. While this statement provides an overview of the additional reporting requirements for factorial trial protocols, we recommend this checklist be used in conjunction with the forthcoming explanation and elaboration document, which provides detailed explanations of each item and examples of good reporting.

This extension checklist represents the minimum essential items for reporting of protocols for factorial trials. For some trials, additional items will be necessary to include in the protocol. For instance, if primary or secondary outcomes differ by factor, this should be reported. Similarly, if multiple testing is thought to be an issue, the protocol should report how this will be handled.

This extension was developed in conjunction with the CONSORT extension for reporting of factorial trials. These 2 extension guidelines provide a framework for cohesive reporting from the trial protocol to final publication of trial results. The latest version of this and other SPIRIT statements can be found online (https://www.spirit-statement.org/).

## Limitations

Although this extension was developed using the best-practice EQUATOR methodological framework, it has some limitations. First, this extension was developed for studies in which results for each factor would be published simultaneously in the same article. This may not always be feasible, for instance when different factors require different sample sizes, or different durations of follow-up. If separate articles are planned to report results from each factor, this should be described in the protocol. Second, although a large and diverse group of stakeholders participated in the Delphi survey, participants were self-selected, which may have affected results. Third, the consensus meeting panelists were chosen based on their expertise and their specific roles relevant to randomized trials (eg, journal editors), and may not be reflective of the views of individuals undertaking factorial trials as a whole. However, the evidence-based approach used to develop this guideline, including a rigorous scoping review of reporting recommendations for factorial trials, may help mitigate the potential effects of these limitations.

## Conclusions

This consensus statement describing an extension of the SPIRIT 2013 Statement provides specific guidance for the reporting of factorial trial protocols. This guidance should help provide greater transparency and completeness in the reporting of these protocols.

## Figures and Tables

**Table 1:** Example of a 2 × 2 Factorial Randomized Trial

Treatment	Factor	Treatment B^a^	
		Active^b^	Control^b^
Treatment A^a^	Active^b^	Active A + active B^c^	Active A + control B^c^
	Control^b^	Control A + active B^c^	Control A + control B^c^

a A and B are factors.

b Active A and control A are levels within factor A; Active-B and Control-B are LEVELS within factor B.

c These items represent the 4 treatment groups. In a full factorial trial all participants are eligible to be randomized among each of the 4 treatment groups; in a partial factorial trial, a subset of participants would only be randomized between active A and control A and automatically assigned to control B without randomization. In a factorial analysis, all participants allocated to intervention A (active A + active-B and active A + control B) are compared against those not allocated to A (control A + active B and control A + control B), and similarly for the comparison for intervention B. In a multiarm analysis, each of the treatment groups is compared against a control (eg, active A + active B, active A + control B, and control A + active B are all compared against control A + control B).

**Table 2:** Glossary of Terms

Term	Definition
Factorial trial	≥2 Interventions assessed in the same participants within a single study
Factor	Includes each intervention and its comparators (eg, factor A is active A and control A)
Level within factors	The specific interventions within a factor (eg, active A and control A are the 2 levels of factor A)
Treatment group	The unique combinations of factors and levels to which participants can be randomized (eg, active A + active Bis 1 treatment group)
Full factorial design	All participants are randomized among all combinations of factors and levels
Partial factorial design	Some participants are not randomized to certain factors
Fractional factorial design	Some combinations of factors are omitted
Comparison	Which treatment groups will be compared against each other
Main comparisons	The comparisons that will primarily be used to draw conclusions about effectiveness of each intervention
Estimand	A description of the treatment effect to be estimated from the trial
Factorial analysis	Also called an *at-the-margins analysis*; all participants allocated to active A are compared against all those allocated to control A, and similarly for the factor B comparison
Multiarm analysis	Also called an *inside-the-table analysis;* the treatment groups (eg, active A + control B, control A + active B) are compared against each other
Interaction	Interactions occur when the effect of one treatment depends on whether participants also receive the other treatment

**Table 3:** Checklist for Reporting of Factorial Randomized Trials: Extension of the SPIRIT 2013 Statement^a^,^b^

Section/topic		Item No.	SPIRIT 2013 checklist item	Extension for factorial trials
**Administrative information**				
Title		1	Descriptive title identifying the study design, population, interventions, and, if applicable, trial acronym	Descriptive title identifying the study as a factorial randomized trial, as well as the population, interventions, and, if applicable, trial acronym
Trialregistration		2a	Trial identifier and registry name. If not yet registered, name of intended registry	
		2b	All items from the World Health Organization Trial Registration Data Set	
Protocol version		3	Date and version identifier	
Funding		4	Sources and types of financial, material, and other support	
Roles and responsibilities		5a	Names, affiliations, and roles of protocol contributors	
		5b	Name and contact information for the trial sponsor	
		5c	Role of study sponsor and funders, if any, in study design; collection, management, analysis, and interpretation of data; writing of the report; and the decision to submit the report for publication, including whether they will have ultimate authority over any of these activities	
		5d	Composition, roles, and responsibilities of the coordinating center, steering committee, endpoint adjudication committee, data management team, and other individuals or groups overseeing the trial, if applicable (see item 21a for data monitoring committee)	
**Introduction**				
Background and rationale		6a	Description of research question and justification for undertaking the trial, including summary of relevant studies (published and unpublished) examining benefits and harms for each intervention	Description of research question and justification for undertaking the trial, including summary of relevant studies (published and unpublished) examining benefits and harms for each intervention, and rationale for using a factorial design, including whether an interaction is hypothesized
		6b	Explanation for choice of comparators	
Objectives		7	Specific objectives or hypotheses	Specific objectives or hypotheses and a statement of which treatment groups form the main comparisons^b^
Trial design		8	Description of trial design including type of trial (eg, parallel group, crossover, factorial, single group), allocation ratio, and framework (eg, superiority, equivalence, noninferiority, exploratory)	Description of the type of factorial trial (eg, full or partial, number of factors, levels within each factor), allocation ratio, and framework (eg, superiority, equivalence, noninferiority, exploratory)
**Methods**				
Participants, interventions, and outcomes				
	Study setting	9	Description of study settings (eg, community clinic, academic hospital) and list of countries where data will be collected. Reference to where list of study sites can be obtained	
	Eligibility criteria	10	Inclusion and exclusion criteria for participants. If applicable, eligibility criteria for study centers and individuals who will perform the interventions (eg, surgeons, psychotherapists)	Inclusion and exclusion criteria for each factor, noting any differences if applicable. If applicable, eligibility criteria for study centers and individuals who will perform the interventions (eg, surgeons, psychotherapists)
	Interventions	11a	Interventions for each group with sufficient detail to allow replication, including how and when they will be administered	
		11b	Criteria for discontinuing or modifying allocated interventions for a given trial participant (eg, drug dose change in response to harms, participant request, or improving/worsening disease)	
		11c	Strategies to improve adherence to intervention protocols, and any procedures for monitoring adherence (eg, drug tablet return, laboratory tests)	
		11d	Relevant concomitant care and interventions that are permitted or prohibited during the trial	
	Outcomes	12	Primary, secondary, and other outcomes, including the specific measurement variable (eg, systolic blood pressure), analysis metric (eg, change from baseline, final value, time to event), method of aggregation (eg, median, proportion), and time point for each outcome. Explanation of the clinical relevance of chosen efficacy and harm outcomes is strongly recommended	
	Participant timeline	13	Time schedule of enrolment, interventions (including any run-ins and washouts), assessments, and visits for participants. A schematic diagram is highly recommended (see Figure)	
	Sample size	14	Estimated number of participants needed to achieve study objectives and how it was determined, including clinical and statistical assumptions supporting any sample size calculations	Estimated number of participants needed to achieve study objectives and how it was determined for each main comparison, including clinical and statistical assumptions supporting any sample size calculations, such as whether an interaction was assumed in the calculation
	Recruitment	15	Strategies for achieving adequate participant enrolment to reach target sample size	
Assignment of interventions (for controlled trials)				
	Sequence generation	16a	Method of generating the allocation sequence (eg, computer-generated random numbers), and list of any factors for stratification. To reduce predictability of a random sequence, details of any planned restriction (eg, blocking) should be provided in a separate document that is unavailable to those who enroll participants or assign interventions	Method of generating the allocation sequence (eg, computer-generated random numbers), list of any variables for stratification, and whether participants were allocated to factors at different time points, if applicable. To reduce predictability of a random sequence, details of any planned restriction (eg, blocking) should be provided in a separate document that is unavailable to those who enroll participants or assign interventions
	Allocation concealment mechanism	16b	Mechanism of implementing the allocation sequence (eg, central telephone; sequentially numbered, opaque, sealed envelopes), describing any steps to conceal the sequence until interventions are assigned	
	Implementation	16c	Who will generate the allocation sequence, who will enroll participants, and who will assign participants to interventions	
	Blinding (masking)	17a	Who will be blinded after assignment to interventions (eg, trial participants, care providers, outcome assessors, data analysts), and how	
		17b	If blinded, circumstances under which unblinding is permissible, and procedure for revealing a participant’s allocated intervention during the trial	
Data collection, management, and analysis				
	Data collection methods	18a	Plans for assessment and collection of outcome, baseline, and other trial data, including any related processes to promote data quality (eg, duplicate measurements, training of assessors) and a description of study instruments (eg, questionnaires, laboratory tests) along with their reliability and validity, if known. Reference to where data collection forms can be found, if not in the protocol	
		18b	Plans to promote participant retention and complete follow-up, including list of any outcome data to be collected for participants who discontinue or deviate from intervention protocols	
	Data management	19	Plans for data entry, coding, security, and storage, including any related processes to promote data quality (eg, double data entry; range checks for data values). Reference to where details of data management procedures can be found, if not in the protocol	
	Statisticalmethods	20a	Statistical methods for analyzing primary and secondary outcomes. Reference to where other details of the statistical analysis plan can be found, if not in the protocol	Statistical methods for each main comparison for primary and secondary outcomes, including: Whether the target treatment effect for each main comparison pertains to the effect in the presence or absence of other factors; Approach, such as factorial or multiarm; How the approach will be chosen, such as pre-specified or based on estimated interaction; If factorial approach to analysis will be used, whether factors will be adjusted for each other; Method(s) for evaluating statistical interactions, and which outcomes (in addition to the primary) they will be applied to; If applicable, how non-concurrent recruitment to factors will be handled; and Reference to where other details of the statistical analysis plan can be found, if not in the protocol
		20b	Methods for any additional analyses (eg, subgroup and adjusted analyses)	
		20c	Definition of analysis population relating to protocol non-adherence (eg, as randomized analysis), and any statistical methods to handle missing data (eg, multiple imputation)	
Monitoring				
	Data monitoring	21a	Composition of data monitoring committee (DMC); summary of its role and reporting structure; statement of whether it is independent from the sponsor and competing interests; and reference to where further details about its charter can be found, if not in the protocol. Alternatively, an explanation of why a DMC is not needed	
		21b	Description of any interim analyses and stopping guidelines, including who will have access to these interim results and make the final decision to terminate the trial	Description of any interim analyses and stopping guidelines, noting any differences across main comparisons, with reasons, and who will have access to these interim results and make the final decision to terminate the trial
	Harms	22	Plans for collecting, assessing, reporting, and managing solicited and spontaneously reported adverse events and other unintended effects of trial interventions or trial conduct	
	Auditing	23	Frequency and procedures for auditing trial conduct, if any, and whether the process will be independent from investigators and the sponsor	
**Ethics and dissemination**				
Research ethics approval		24	Plans for seeking research ethics committee/institutional review board (REC/IRB) approval	
Protocol amendments		25	Plans for communicating important protocol modifications (eg, changes to eligibility criteria, outcomes, analyses) to relevant parties (eg, investigators, REC/IRBs, trial participants, trial registries, journals, regulators)	
Consent or assent		26a	Who will obtain informed consent or assent from potential trial participants or authorized surrogates, and how (see item 32)	
		26b	Additional consent provisions for collection and use of participant data and biological specimens in ancillary studies, if applicable	
Confidentiality		27	How personal information about potential and enrolled participants will be collected, shared, and maintained in order to protect confidentiality before, during, and after the trial	
Declaration of interests		28	Financial and other competing interests for principal investigators for the overall trial and each study site	
Access to data		29	Statement of who will have access to the final trial data set and disclosure of contractual agreements that limit such access for investigators	
Ancillary and post-trial care		30	Provisions, if any, for ancillary and post-trial care, and for compensation to those who suffer harm from trial participation	
Dissemination policy		31a	Plans for investigators and sponsor to communicate trial results to participants, health care professionals, the public, and other relevant groups (eg, via publication, reporting in results databases, or other data sharing arrangements), including any publication restrictions	
		31b	Authorship eligibility guidelines and any intended use of professional writers	
		31c	Plans, if any, for granting public access to the full protocol, participant-level data set, and statistical code	
**Appendices**				
Informed consent materials		32	Model consent form and other related documentation given to participants and authorized surrogates	
Biological specimens		33	Plans for collection, laboratory evaluation, and storage of biological specimens for genetic or molecular analysis in the current trial and for future use in ancillary studies, if applicable	

Abbreviations: IRB, institutional review board; REB, research ethics board; SPIRIT, Standard Protocol Items: Recommendations for Interventional Trials.

a It is recommended that this checklist is read in conjunction with the SPIRIT 2013 Statement^1^ for important clarification on the items.

b Each overall intervention group to be compared is a factor (eg, active A and control A together are 1 factor; active B and control B together are another factor). The specific interventions within a factor are the levels (eg, active A and control A are the 2 levels of factor A). Treatment groups are the unique combinations of factors and levels (eg, in a 2 × 2 trial with factors A and B, there will be 4 treatment groups, eg, active A + control B, active A + active B). The main comparison is which treatment groups will be compared against each other to draw main conclusions about the effectiveness of each intervention.

## Box. An Overview of Estimands in Factorial Trials

### Estimands for Factorial Trials

- Estimands are used to describe the research questions a trial aims to address.
- In factorial trials, different types of estimands can be specified depending on the aims.
- For 2-in-1 trials, estimands are typically based around the comparison of treatment A vs not A (and similarly for other factors). However, this estimand can be defined in different ways; for instance, it could be based on the comparison of treatment A vs not A if no one received treatment B, or as the effect of A vs not A if everyone received treatment B.
- Alternatively, the estimand for treatment A could be defined based on the comparison of A vs not A averaged across those who do and those who do not receive treatment B.^a^ However, this estimand does not typically reflect how treatments are used in practice, and so other estimands are usually more relevant for 2-in-1 trials.
- For trials aiming to determine whether treatments interact, the estimand may be based around the difference in the effects of treatment A if no one received treatment B vs if everyone received treatment B.

### Implications for Statistical Analysis

- The method of statistical analysis should be chosen based on the estimand.
- For 2-in-1 trials, a factorial (also known as *at-the-margins*) analysis is typically used due to its efficiency. However, this analysis averages across the 2 strata of those allocated to receive and not receive B, and so it only estimates the effect of treatment A if no one receives B if treatments A and B do not interact. If treatments do interact, it estimates an average effect of A across the strata of B, which is not usually of primary interest.
- A multiarm (also known as *inside-the-table*) analysis can also estimate the effect of treatment A if no one receives B, even when treatments A and B do interact. However, because it is less efficient than the factorial analysis, it is less frequently used for 2-in-1 trials.

a This could correspond either to some proportions defined by investigators, or else to the study proportions allocated to B and not B. Therefore, the exact method of averaging should be made explicit. If this average is defined based on the study proportions, it should be clarified whether this is based on the initially specified allocation ratio (eg, 1:1), or the final observed proportions in each stratum. These may differ substantially if, for instance, randomization to factor B is stopped partway through the trial for safety reasons.

## Key Points

### Question

What additional information should be provided in protocols of factorial randomized trials?

### Findings

This consensus statement provides an extension of the Standard Protocol Items: Recommendations for Interventional Trials (SPIRIT) 2013 Statement. Nine SPIRIT items have been modified.

### Meaning

This SPIRIT extension checklist can facilitate transparent reporting of factorial trial protocols and may help enhance trial utility.

## Data Sharing Statement

See the Supplement.
